# Antibacterial activity and mechanism analysis of deoxycholic acid against Clostridium perfringens

**DOI:** 10.21203/rs.3.rs-7454314/v1

**Published:** 2025-11-05

**Authors:** Xinglong Song, Qingyun Guo, Zhenyu Zhong, Jiade Bai, Meihui Wang, Congshan Yang, Qingxun Zhang

**Affiliations:** National Conservation and Research Center for Milu; National Conservation and Research Center for Milu; National Conservation and Research Center for Milu; National Conservation and Research Center for Milu; National Conservation and Research Center for Milu; Anhui Agricultural University; National Conservation and Research Center for Milu

**Keywords:** Clostridium perfringens, Deoxycholic acid, Antimicrobial activity, Transcriptomics

## Abstract

**Background:**

Deoxycholic acid (DCA), a gut microbiota-derived secondary metabolite, exhibits broad-spectrum antimicrobial activity, particularly against *Clostridium perfringens*(*C. perfringens*). However, its precise mechanistic action remains unclear.

**Objective:**

In this study, we examined the inhibitory mechanism of DCA against *C. perfringens* through in vitro growth inhibition assays coupled with transcriptomic analysis.

**Result:**

The study results indicate that DCA can effectively inhibit the formation of *C. perfringens* biofilms, disrupt their cell walls, increase cell membrane permeability, and cause nucleic acid leakage. Transcriptome analysis revealed that DCA can mediate its oxidative damage by up-regulating the oxidative phosphorylation pathway in *C. perfringens* and down-regulating antioxidant activity, peroxidase activity, and oxidoreductase activity. This study provides insights into the antimicrobial activity of DCA and its mechanisms, laying a theoretical foundation for its development as a novel antimicrobial agent or feed additive.

## Introduction

*C. perfringens*, a gram-positive anaerobic bacterium, is widely found in the intestinal microbiota of humans and animals, in water bodies, and in soil. It significantly impairs animal health and production performance, resulting in substantial annual economic losses to the global livestock industry[1–3]. Furthermore, *C. perfringens* is an important zoonotic pathogen. It not only endangers the health of animals but can also be transmitted to humans through contaminated food, leading to public health issues such as food poisoning. The Centers for Disease Control and Prevention, USA, reported that approximately 48 million Americans fall ill each year due to food poisoning caused by *C. botulinum* infection. In some developing and relatively poor countries, the public health risk posed by *C. perfringens* is even more serious[4, 5].

Treatment for *C. perfringens* infections typically involves antibiotics; however, *C. perfringens* has developed resistance to multiple antimicrobial agents, including chloramphenicol, lincosamides, tetracyclines, aminoglycosides, and macrolides[6–8]. Notably, β-lactams and oxazolamides are the drugs of choice for treating *C. perfringens*, but strains resistant to these drugs have now emerged. [8, 9]. Plasmids have mediated the evolution and rapid adaptation of these drug-resistant strains, posing a major threat to veterinary health, food safety, and public health[10]. Consequently, there is an urgent need to develop novel antimicrobial agents or antibacterial strategies to escalating issue of antimicrobial resistance in *C. perfringens*.

In recent years, the significant role of gut microbiota-derived metabolites in enhancing host health and regulating immune function has been widely confirmed. These metabolites, known as postbiotics, include exopolysaccharides (EPS), short chain fatty acids (SCFAs), bile acids (BAs), and other[11]. DCA is a secondary bile acid derived from the conversion of hepatocyte-synthesized primary bile acid by intestinal flora upon entering the intestine[12, 13]. While previous research on DCA primarily centered on its involvement in liver and gastrointestinal diseases[14, 15], recent studies have revealed its broad-spectrum antibacterial activity. DCA exhibits antimicrobial activity against pathogens such as *Klebsiella pneumoniae, Streptococcus pneumoniae*, *Staphylococcus aureus*, and *C. perfringens*. It can regulate the intestinal microbiota and alleviate the inflammatory response triggered by these pathogens, thereby making it a potential antimicrobial drug[16–19].

Studies have shown that deoxycholic acid has a strong specific antibacterial effect on *C.perfringens*, and can alleviate necrotic enteritis in chickens caused by *C. perfringens* through multiple pathways, including attenuating inflammatory cyclooxygenase signaling and enhancing host defense peptide synthesis[19, 20]. However, its antibacterial mechanism of DCA remains incompletely understood. Considering the broad-spectrum antimicrobial properties of DCA, this study aims to systematically evaluate its in vitro antibacterial activity against *C. perfringens* and investigate the underlying inhibitory mechanisms. The research results are expected to provide theoretical reference for DCA as a new antibacterial agent against *C. perfringens* or as a feed additive in animal husbandry.

## Materials and methods

### Bacterial and antimicrobial agents

*C. perfringens* (CVCC2015) type A as an experimental strain. Bacteria were cultured using Tryptic Sulfite Cycloserine (TSC) agar and Fluid Thioglycollate Medium (FTG) under anaerobic conditions. DCA was purchased from Beijing Solarbio Technology Co., Ltd, purity > 99%.

#### Minimum inhibitory concentration (MIC) and Minimum bacterial concentration (MBC) of DCA against C. perfringens

The minimum inhibitory concentration (MIC) of DCA against *C. perfringens* was determined using the broth microdilution method in a 96-well plate. The MIC value was defined as the lowest concentration required to inhibit bacterial growth in the 96-well plate. At the MIC concentration, bacterial growth is inhibited but the bacteria are not killed in the culture medium. Subsequently, we spread the culture medium from the 96-well plate at concentrations above the MIC onto tryptic sulfate cycloserine (TSC) agar medium, and the concentration at which no bacterial growth was observed on the agar was defined as the MBC [21].

#### Effect of DCA on the growth curve of C. perfringens

*C. perfringens* was inoculated in FTG broth supplemented with DCA at concentrations of 1/4, 1/2 and 1 MIC, and the same volume of PBS was added to the control group. Bacteria were collected every hour to measure OD600_nm_. Each concentration treatment group was set up in three times.

### Assessment of cell wall integrity and cell membrane permeability

Currently, alkaline phosphatase (AKP) and β-galactosidase (β-gal) have become important indicators for determining the integrity of bacterial cell walls and cell membranes[22, 23]. AKP is located between the bacterial cell wall and the cell membrane. Upon disruption of the cell wall, AKP is released into the extracellular environment[5]. Similarly, alterations in cell membrane permeability can be assessed by measuring the intracellular release of β-gal in *C. perfringens*. After 6 hours of cultivation, the bacterial concentration was adjusted to an optical density (OD) of 0.5 at 600 nm. Subsequently, *C. perfringens* was exposed to different concentrations of DCA, with PBS serving as the negative control. After treatment, the supernatant was collected by centrifugation, and the levels of AKP and β-gal were measured to assess the extent of damage to the bacterial cell wall and membrane. At the same time, the concentration of dsDNA in the supernatant was measured using an ultra-micro ultraviolet spectrophotometer to observe the level of intracellular nucleic acid leakage.

#### Effect of DCA on the biofilm of C. perfringens

The impact of DCA on the biofilm formation of *C. perfringens* was assessed utilizing the crystal violet assay[24]. FTG broth, supplemented with varying concentrations of DCA, was aliquoted into 96-well microtiter plates, with each concentration being triplicated across three rows. Subsequently, *C. perfringens* was then inoculated into the wells and incubated overnight to facilitate biofilm formation. Following incubation, the bacterial suspension in the wells was removed, and the plates were gently washed twice with PBS to remove non-adherent bacteria and residual culture medium. After drying, 100 μL of anhydrous methanol was added to each well to fix the biofilm for 15 minutes. Subsequently, 200 μL of 1% crystal violet solution was applied to stain the wells for 30 minutes. Then wash the plate with PBS and dissolve the biofilm with 33% glacial acetic acid for 10 minutes. Finally, the absorbance at OD570_nm_ was measured. The absorbance value was directly proportional to the biofilm mass, thereby reflecting the effect of DCA on the formation of *C. perfringens* biofilm.

### Transmission electron microscopy (TEM) and Scanning electron microscopy (SEM) inspection

The concentration of *C. perfringens* cultured for 6 hours was adjusted to OD600_nm_ of 0.5. The bacterial precipitate was subsequently inoculated with DCA solution at concentrations of 0, 1 MIC and 8 MIC, and co-cultured for 2 hours at 37°C. Following incubation, the bacteria were collected by centrifugation, gently rinsed with PBS, and the PBS was discarded. The 2.5% glutaraldehyde electron microscopy fixative was added, and the bacteria were resuspended in the fixative for two hours at room temperature. The samples were processed according to a previously published protocol and subsequently examined using TEM and SEM[25, 26].

### Total RNA extraction and RNA sequencing

#### Total RNA extraction and RNA sequencing

Four independent samples of the DCA-treated group (1 MIC) and the control group of *C. perfringens* were cultured for 6 h at 37°C. Total RNA was extracted from bacterial precipitates, and the extracted RNA was quality assessed to ensure its integrity, concentration, and purity. Libraries were constructed using the TruSeq^™^ Stranded Total RNA Library Prep Kit with rRNA depletion. NovaSeqXPlus sequencing platform (ShangHai majorbio Bio-pharm technology Co.,ltd) was used for mRNA sequencing.

### Analysis of differentially expressed genes

The EBSeq algorithm was used to screen the differentially expressed genes, and the criteria for screening were Fold Change (FC) greater than 1.5 and FDR < 0.05. The differentially expressed genes were annotated in the GO (Gene Ontology) database and KEGG (Kyoto Encyclopedia of Genes and Genomes) database, and the enrichment analysis was carried out; the significance levels of the GO and KEGG Pathways enriched with differentially expressed genes were analysed by the Fisher exact test based on the hypergeometric distribution. KEGG Pathway significance levels, and screened out the GO items and metabolic pathways that were significantly enriched for differentially expressed genes.

### qPCR for Detection of Gene Expression

For validation of transcriptional profiling from RNA-Seq, quantitative real-time PCR (qRT-PCR) was performed. Total RNA was reverse transcribed into cDNA using the PrimeScript RT reagent kit (Takara, Japan). qPCR was performed with ChamQ SYBR qPCR Master Mix (High ROX Premixed) (Vazyme, Nanjing, China). Primers for qPCR were obtained by querying the gene database at NCBI and were synthesized by Bioengineering Biotechnology Co. (Bioengineering Biotechnology, Shanghai, China). The relative expression level of genes was represented by 2^(−ΔΔCt), and 16sRNA was used as a reference gene[22]. The primer details are shown in **supplementary table S1**.

### Statistical analysis

All the experimental data were replicated three times or more, statistically analyzed by SPSS Statistics 26.0, and plotted by GraphPad Prism 9.0. The results of the experiments were expressed as the ‘mean ± standard deviation’, and the t-test was used to compare the differences between the groups, with * representing a significant difference (*p* < 0.05) and ** representing a highly significant difference (*p* < 0.01).

## Result

### Effect of DCA on the growth curve and biofilm of C. perfringens

**Figure 1:**
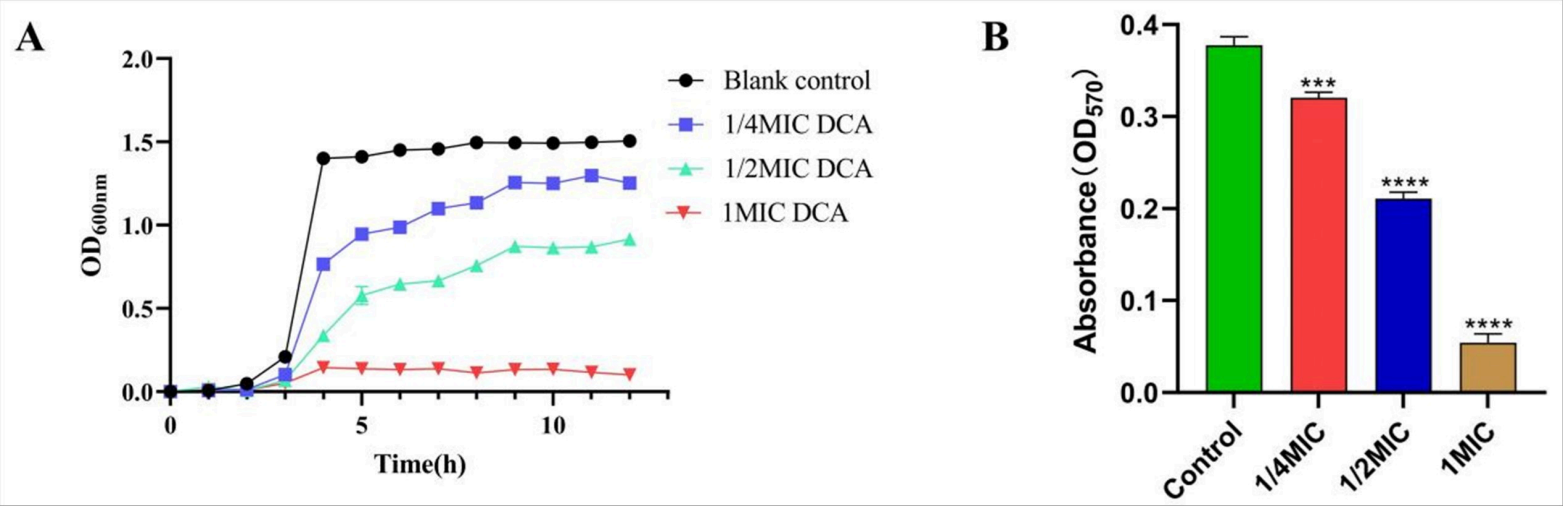
The inhibitory effect of DCA on the growth and biofilm formation of *C. perfringens*. **A** Effect of different concentrations of DCA on the growth curve of *C. perfringens*. **B** Effect of DCA on biofilm formation of *C. perfringens*, OD570_nm_ indicates biofilm content.

The results showed that DCA had a strong inhibitory effect on *C. perfringens* with a MIC value of 0.125 mg/mL and MBC value of 1 mg/mL (8 MIC). As is shown in Fig. 1A, when the DCA concentration is 1/4 MIC and 1/2 MIC, it can significantly inhibit the growth of *C. perfringens*. When the concentration is 1 MIC, DCA can completely inhibit the growth of *C. perfringens*. As is shown in Fig. 1B, the results of the crystal violet assay showed that DCA at concentrations ranging from 1/4 MIC to 1 MIC significantly inhibited the formation of *C. perfringens* biofilm in a concentration-dependent manner compared with the control group.

### Effect of DCA on the membrane integrity of C. perfringens cell walls

**Figure 2:**
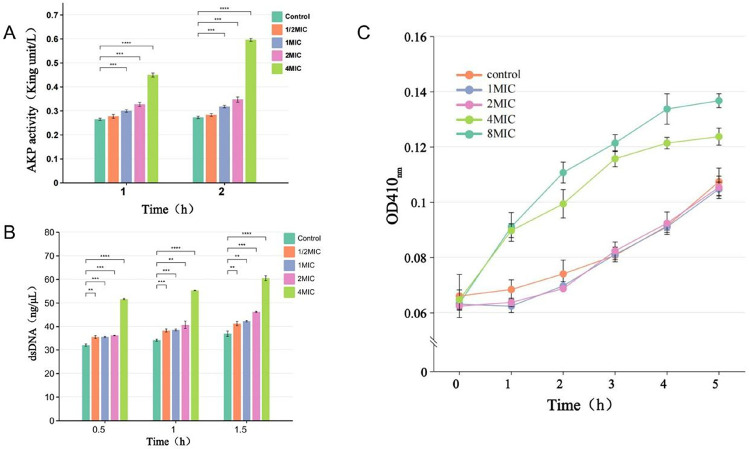
The effect of DCA on the cell membrane permeability and cell wall integrity of *C. perfringens*. **A** Effect of different concentrations of DCA treatment on cell membrane integrity of *C. perfringens*, OD415 nm represents extracellular β-Gal content, and higher values indicate more severe cell membrane disruption. **B** Extracellular AKP content in *C. perfringens* after treatment with different concentrations of DCA, a value that indirectly reflects cell wall integrity. **C**Extracellular DNA concentration of *C. perfringens* after treatment with different concentrations of DCA.

From the detection of AKP and β-gal content (Fig. 2A, Fig. 2C), DCA can destroy the integrity of cell walls and the permeability of cell membranes, and this destructive ability is proportional to the concentration of DCA. As shown in Fig. 2B, the concentration of extracellular dsDNA significantly increased after DCA treatment, indicating that DCA can alter cell membrane permeability, leading to nucleic acid leakage, which may have a negative impact on maintaining normal bacterial physiological functions.

### Effect of DCA on C. perfringens morphology

**Figure 3:**
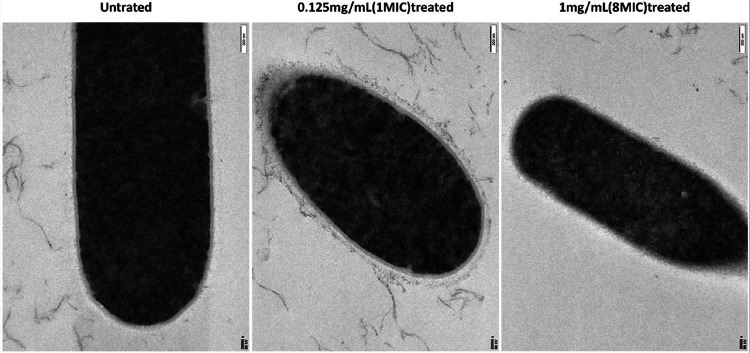
TEM micrographs of *C. perfringens*treated with DCA.

**Figure 4:**
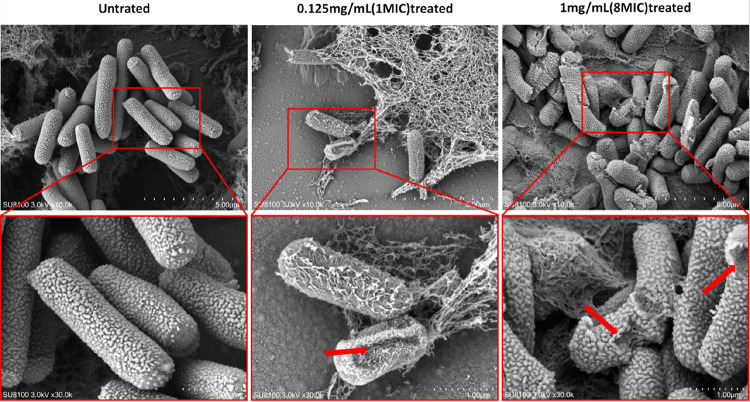
SEM micrographs of *C. perfringens*treated with DCA at the MIC/MBC (8MIC) and control cells without treatment.

TEM and SEM further showed the disruptive effect of DCA on the structure of *C. perfringens*. As can be seen in Fig. 3 and Fig. 4, the untreated cells (control) showed a typical *C. perfringens* ultrastructure, with the cell membrane, cell wall and no cellular damage was observed. After treatment with MIC concentration of DCA for 2 hours, some of the cell edges were blurred and the surface of the cell wall appeared wrinkled. Under the treatment with MBC concentration of DCA, the cell wall membrane was completely blurred and the bacterial surface showed a large number of breaks and was no longer intact. Scanning electron microscopy and projection results showed that low concentrations of DCA had a certain destructive effect on the cell membrane and cell wall of *C. perfringens*, while at the concentration of MBC, DCA could directly destroy the cell structure of *C. perfringens* leading to the death of the bacteria.

### Analysis of differentially expressed genes (DEGs)

**Figure 5:**
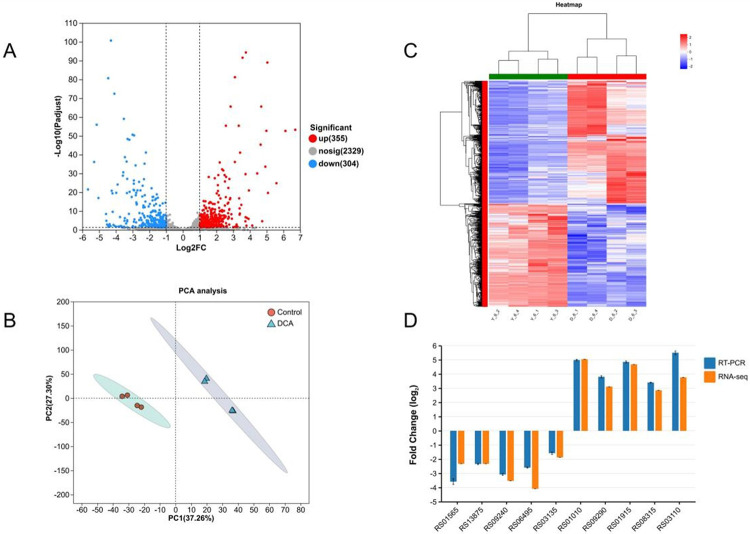
Overview of the gene expression analysis. **A**Volcano mapping of differentially expressed genes (DEGs) based on RNA-seq analysis of *C. perfringens* after untreated and DCA treatment. **B**Principal component analysis (PCA) of *C. perfringens* gene expression in the DCA treated and control groups. **C** Hierarchical clustering heat map of different gene expressions in different experimental conditions. Red represents high gene expression and blue represents low gene expression. **D**Comparison of fold changes in DEGs between RNA-seq and RT-PCR methods.

Transcriptomics analysis was used to understand the gene expression changes between DCA-treated and untreated groups. A total of 659 DEGs were identified compared to the control group of these, 355 were up-regulated and 304 were down-regulated (Fig. 5A). Details of the DEGs are shown in **supplementary table S2**.

**Figure 6:**
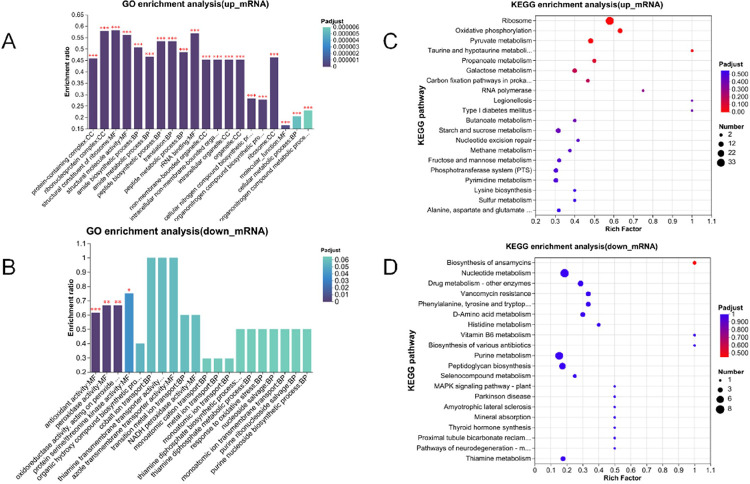
DEGs KEGG and GO enrichment ranking results. **A**GO enrichment analysis of up-regulated genes in *C. perfringens* after DCA treatment. **B** GO enrichment analysis of down-regulated genes in *C. perfringens* after DCA treatment. **C** KEGG enrichment analysis of up-regulated genes in *C. perfringens* after DCA treatment. **D** KEGG enrichment analysis of down-regulated genes in *C. perfringens* after DCA treatment.

To further explore the effect of DCA on *C. perfringens*, the differentially expressed genes were subjected to GO functional gene enrichment analysis. As seen in Fig. 6A, GO enrichment results showed that 202 GO terms were up-regulation, mainly involving protein-containing complex, ribonucleoprotein complex, structural constituent of ribosome and structural molecule activity. The down-regulated GO terms are enriched in molecular function, specifically enriched in four GO terms: antioxidant activity, peroxidase activity, oxidoreductase activity and protein serine/threonine kinase activity(Fig. 6B). While Details of GO enrichment of DEGs are shown in **supplementary table S3**.

The differentially expressed genes of *C. perfringens* after DCA were subjected to KEGG enrichment analysis, and as shown in Fig. 6C, after DCA treatment, up-regulated genes were enriched in four pathways: Ribosome, oxidative phosphorylation, pyruvate metabolism and taurine and hypotaurine metabolism. Down-regulated genes were enriched in pathways that did not show significant differences (Fig. 6D). See **supplementary table S4** for details.

## Discussion

The spores of *C. perfringens* exhibit strong environmental resistance, capable of withstanding extreme conditions such as high temperatures and dryness, and demonstrate significant resistance to conventional chemical disinfectants[27]. Additionally, with the widespread use of antibiotics, this strain has gradually evolved to develop multidrug resistance. To reduce antibiotic use and mitigate the threat this bacterium poses to public health, the development of novel antimicrobial agents to address *C. perfringens* infections has become an urgent research priority. Currently, there is very little research on non-antibiotic antimicrobial drugs for *C. perfringens*. Zheng et al. studied the antibacterial activity of natural propolis and identified caffeic acid as its primary component against *C. perfringens*, concluding that propolis could serve as an alternative to antibiotics[5]. DCA was proposed in 2019 as a potential treatment for necrotic enteritis (NE) caused by *C. perfringens* in chickens[20]. Subsequently, Kim et al. conducted further research and found that DCA can induce the production of host defense peptides to alleviate NE, further revealing the potential of DCA as an antibiotic alternative[19]. However, these two studies focused on the effects of DCA on the host, and there have been no studies on how DCA acts on *C. perfringens*. Therefore, the aim of our study was to reveal the antibacterial activity and mechanism of DCA against *C. perfringens*.

By detecting AKP and β-gal activity, we can observe that DCA can disrupt the cell wall and cell membrane structure of *C. perfringens*. To validate these results, we directly observed the cellular structure using SEM and TEM. The control group of *C. perfringens* exhibited a thick, mature, and dense three-dimensional structure. At a DCA concentration of 1 MIC, the cell wall already showed significant wrinkling and deformation, and at an 8 MIC concentration, the cells began to rupture, with intracellular material leaking out, indicating that DCA can effectively disrupt the cellular structure of *C. perfringens*.

The virulence and drug resistance of *C.perfringens* is closely related to the formation of biofilms, which can prevent or delay the penetration of antibiotics and allow them to escape the killing effect of antimicrobial drugs[28]. As can be seen from Fig. 1D, DCA can significantly inhibit the formation of *C. perfringens biofilms*, and transcriptomic results indicated that DCA treatment significantly down-regulated the expression of the *spoVG* gene in *C. perfringens* (log_2_FC=−3.00). The *spovG* gene is widely present in bacteria and has been confirmed to be a key regulatory factor in biofilm formation in bacteria such as *Bacillus subtilis*, *Listeria monocytogenes*, *Staphylococcus aureus*, *Bacillus cereus*, and *Staphylococcus epidermidis*[28–30]. However, whether the *spoVG* gene can regulate the formation of *C. perfringens* biofilms has not yet been confirmed and requires further research.

Among the DEGs, *def* gene was significantly down-regulated(log2FC=−3.52), the down-regulation of this gene was also verified by qPCR(RS09240). The *def* gene encodes peptide deformylase (PDF), an enzyme whose core function is to catalyze the removal of the formyl group (-CHO) from the N-terminal formylmethionine of newly synthesized polypeptide chains, thereby forming methionine. This is an important step in post-translational modification of bacterial proteins. If PDF is inhibited, it leads to the accumulation of un-deformylated proteins, resulting in bacterial growth arrest or death[31–33]. This enzyme, which is widely present in bacteria, has become an important target for the development of new antimicrobial drugs[34, 35]. This indicates that DCA exerts its antibacterial effect against *C. perfringens* by suppressing bacterial protein synthesis through down-regulation of the *def* gene.

Transcriptomics is important for understanding the antibacterial mechanism of DCA. KEGG enrichment results showed that the oxidative phosphorylation pathway was significantly enriched. Oxidative phosphorylation is the main pathway for ATP production in bacteria, and up-regulation of this pathway promotes the activity of the bacterial electron transport chain; however, the high activity of the ETC may lead to electron leakage, which reacts with oxygen to generate large amounts of reactive oxygen species (ROS), such as superoxide anions and hydrogen peroxide[36]. GO enrichment analysis showed that antioxidant activity, peroxidase activity and oxidoreductase activity were down-regulated in *C. perfringens*. These enzymes are normally responsible for scavenging ROS from the cell, and if the activity of these enzymes is reduced, the ability of the bacteria to process ROS is diminished, leading to ROS accumulation[37, 38]. This indicates that DCA can induce ROS by upregulating the oxidative phosphorylation pathway. At the same time, the bacterial antioxidant system is inhibited and unable to effectively clear these ROS, resulting in an increase in intracellular ROS levels and triggering oxidative stress. Excessive ROS can damage bacterial DNA, proteins, and lipids, leading to the destruction of cellular structures and ultimately causing cell death[39, 40].

## Conclusions

In conclusion, DCA exhibits potent antibacterial activity against *C. perfringens*, effectively inhibiting biofilm formation, disrupting bacterial cell wall integrity, and increasing membrane permeability. Transcriptome results indicate that DCA mediates oxidative damage by upregulating the oxidative phosphorylation pathway in *C. perfringens* and downregulating antioxidant activity, peroxidase activity, and oxidoreductase activity. Additionally, DCA suppresses the expression of the *def* gene, thereby impairing bacterial protein synthesis. This multi-targeted mechanism underscores DCA’s broad-spectrum antibacterial efficacy, making it a promising candidate for combating *C. perfringens* infections.

## Supplementary Material

Supplementary Files

This is a list of supplementary files associated with this preprint. Click to download.

- supplementarytableS3.csv
- supplementarytableS4.csv
- supplementarytableS1.csv
- supplementarytableS2.csv

## Data Availability

The datasets generated and analyzed during the present study can be obtained from the first author upon reasonable request. The raw sequencing data generated in this study have been deposited in the NCBI Sequence Read Archive (SRA) under BioProject accession number PRJNA1314926. https://dataview.ncbi.nlm.nih.gov/?search=SUB15589106&archive=bioproject. Other data are provided in the supplementary information file.
